# Supplementary material for: Serum-Free Media Formulation Using Marine Microalgae Extracts and Growth Factor Cocktails for Madin-Darby Canine Kidney and Vero Cell Cultures
Source: Int J Mol Sci. 2024 Sep 12;25(18):9881. doi: 10.3390/ijms25189881 (PMC11432547; doi:10.3390/ijms25189881)
Supplement: Supplementary file 1 [file ijms-25-09881-s001.zip › ijms-3189123-supplementary.pdf]

Supplemental Materials to the paper

Serum-Free Media Formulation Using Marine Microalgae Extracts and Growth Factor Cocktails for Madin-Darby canine kidney and Vero Cell Cultures

Areumi Park [1], Yeon-Ji Lee [1], Eunyoung Jo [1], Gun-Hoo Park [1], Seong-Yeong Heo [1,2], Eun-Jeong Koh [1], Seung-Hong Lee [3], Seon-Heui Cha [4], and Soo-Jin Heo [1,2,*]

[1] Jeju Bio Research Center, Korea Institute of Ocean Science and Technology (KIOST), Jeju 63349, Republic of Korea; areumi1001@kiost.ac.kr (A.P.); leeyj0409@kiost.ac.kr (Y.-J.L.); jey8574@kiost.ac.kr (E.J.); gunhoopark@kiost.ac.kr (G.-H.P.); syheo@kiost.ac.kr (S.-Y.H.); kej763@kiost.ac.kr (E.-J.K.)
[2] Department of Marine Technology & Convergence Engineering (Marine Biotechnology), University of Science and Technology (UST), Daejeon 34113, Republic of Korea
[3] Department of Pharmaceutical Engineering, Soonchunhyang University, Asan 31538, Republic of Korea; shlee80@sch.ac.kr
[4] Department of Marine Bio and Medical Sciences, Hanseo University, Seosan-si 32158, Republic of Korea; sunnycha@hanseo.ac.kr
* Correspondence: sjheo@kiost.ac.kr

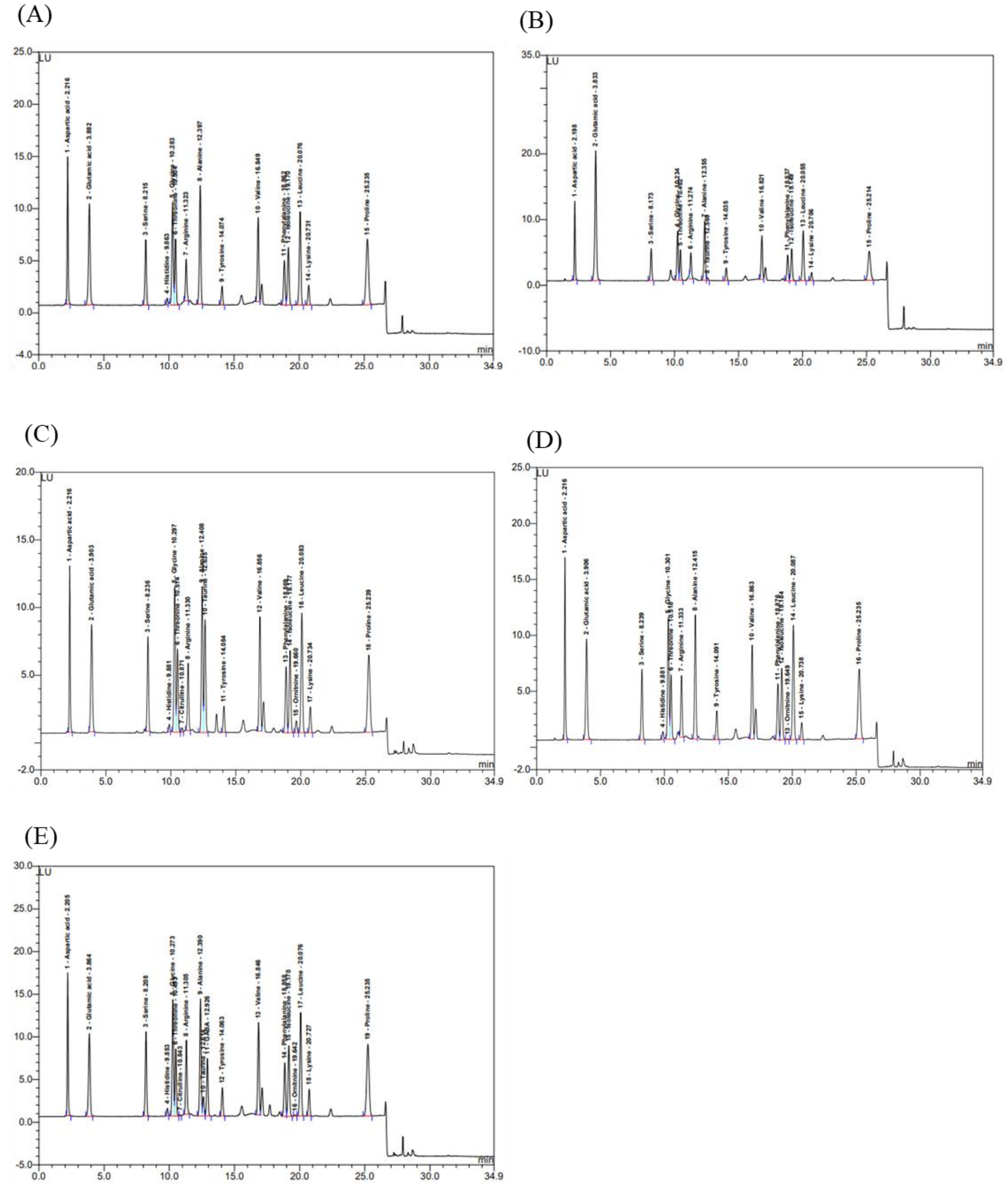

Figure S1. The amino acid HPLC chromatogram of microalgae extract. (A) *Spirulina platensis*, (B) *Dunaliella salina*, (C) *Haematococcus pluvialis*, (D) *Nannochloropsis salina*, (E) *Tetraselmis* sp
